# The Reverse Cholesterol Transport Pathway Improves Understanding of Genetic Networks for Fat Deposition and Muscle Growth in Beef Cattle

**DOI:** 10.1371/journal.pone.0015203

**Published:** 2010-12-03

**Authors:** Tyler F. Daniels, Xiao-Lin Wu, Zengxiang Pan, Jennifer J. Michal, Raymond W. Wright, Karen M. Killinger, Michael D. MacNeil, Zhihua Jiang

**Affiliations:** 1 Department of Animal Sciences, Washington State University, Pullman, Washington, United States of America; 2 Department of Dairy Science, University of Wisconsin-Madison, Madison, Wisconsin, United States of America; 3 School of Food Science, Washington State University, Pullman, Washington, United States of America; 4 USDA-ARS, Fort Keogh Livestock and Range Research Laboratory, Miles City, Montana, United States of America; VU University Medical Center and Center for Neurogenomics and Cognitive Research, VU University, The Netherlands

## Abstract

In the present study, thirteen genes involved in the reverse cholesterol transport (RCT) pathway were investigated for their associations with three fat depositions, eight fatty acid compositions and two growth-related phenotypes in a Wagyu x Limousin reference population, including 6 F_1_ bulls, 113 F_1_ dams, and 246 F_2_ progeny. A total of 37 amplicons were used to screen single nucleotide polymorphisms (SNPs) on 6 F_1_ bulls. Among 36 SNPs detected in 11 of these 13 genes, 19 were selected for genotyping by the Sequenom assay design on all F_2_ progeny. Single-marker analysis revealed seven SNPs in ATP binding cassette A1, apolipoproteins A1, B and E, phospholipid transfer protein and paraoxinase 1 genes significantly associated with nine phenotypes (P<0.05). Previously, we reported genetic networks associated with 19 complex phenotypes based on a total of 138 genetic polymorphisms derived from 71 known functional genes. Therefore, after Bonferroni correction, these significant (adjusted *P*<0.05) and suggestive (adjusted *P*<0.10) associations were then used to identify genetic networks related to the RCT pathway. Multiple-marker analysis suggested possible genetic networks involving the RCT pathway for kidney-pelvic-heart fat percentage, rib-eye area, and subcutaneous fat depth phenotypes with markers derived from paraoxinase 1, apolipoproteins A1 and E, respectively. The present study confirmed that genes involved in cholesterol homeostasis are useful targets for investigating obesity in humans as well as for improving meat quality phenotypes in a livestock production.

## Introduction

Reverse cholesterol transport (RCT) pathway represents an important process involved in cholesterol homeostasis [1]–[2]. In the process, high density lipoproteins (HDL) serve as transport particles by which peripheral cell cholesterol can be returned to the liver for catabolism [3]. Studies have shown that apolipoprotein A1 (APOA1) is an essential co-factor for several key components of RCT including lecithin:cholesterol acyltransferase (LCAT) [4], ATP binding cassette A1 (ABCA1) [5], and scavenger receptor B1 (SCARB1) [6]. Similarly, apolipoprotein B (APOB) is required for synthesis of chylomicrons and very low density lipoprotein (VLDL) in the intestines and the liver [7]–[8]. Meanwhile, apolipoprotein C-II (APOC2) is responsible for activation of lipases, on chylomicrons and VLDLs [9], a crucial aspect of fatty acid homeostasis. Finally, apolipoprotein E (APOE) is extremely important to low density lipoproteins (LDL) and chylomicron remnant clearance through the low density lipoprotein receptor (LDLR) [10]. On the other hand, HDL protects against atherosclerosis primarily via RCT, but also has powerful antioxidant properties directed specifically toward oxidized lipids inside lipoproteins. This effect appears to be caused by the paraoxidase I (*PON1*) gene product [11].

In circulation, the activities of lipoprotein lipase (LPL), endothelial lipase (LIPG), and hepatic lipase (LIPC) continuously remodel lipoproteins, which profoundly affect their metabolic fate. The primary function of LPL is to hydrolyze triglyceride-rich lipoproteins, especially chylomicrons and VLDL, thereby generating free fatty acids and glycerol for energy metabolism and storage [9]. These lipoproteins, along with HDL, are also modified by LIPG, which primarily hydrolyzes phospholipids [12]. LIPC has powerful VLDL and IDL (intermediate density protein) triglyceride hydrolysis capabilities [13], as well as the ability to condense HDL into a subspecies that is more likely to interact with SCARB1 for cholesterol efflux or endocytosis [14]. In addition, the phospholipid transfer protein (PLTP) facilitates the transfer of phospholipids and to a lesser extent, cholesterol from triglyceride rich lipoproteins such as VLDL and chylomicrons into HDL [15]–[16].

All these molecular events indicate that the RCT pathway is a major component of lipid homeostasis affecting lipid phenotypes. Mammals achieve intravascular lipid transport though production and metabolism of lipoproteins, which distribute cholesterol and fatty acids to peripheral tissues expressing the appropriate lipases/receptors. However, little effort has been directed towards understanding how the RCT pathway affects different fat depot storages, fatty acid compositions and overall body growth. Furthermore, differences in dietary intake and digestive physiology between ruminants and non-ruminants produce questions regarding the importance of these pathways to cattle. To this end, the aim of the present study was to investigate the potential molecular links of the RCT pathway genes with the fat deposition, fatty acid composition and body growth-related phenotypes using cattle as a model organism.

## Materials and Methods

### Animals and phenotypes

A Wagyu x Limousin reference population was jointly developed by Washington State University and the Fort Keogh Livestock and Range Research Laboratory, ARS, USDA, as previously described [17]–[18]. The Fort Keogh Livestock and Range Research Laboratory Institutional Animal Care and Use Committee approved all protocols that involved use of animals in this research study. The DNA samples used in the present study were derived from 6 F_1_ bulls, 113 F_1_ dams, and 246 F_2_ progeny in the Fort Keogh Livestock and Range Research Laboratory, ARS, USDA. Fat deposition for three depots was measured by a trained evaluator after 48 h of chilling at 2°C and included beef marbling score (BMS), subcutaneous fat depth (SFD) and percent kidney-pelvic-heart fat (KPH). BMS reflects the dispersion of intramuscular fat in the *longissimus* muscle and is determined subjectively based on U.S. Department of Agriculture standards (http://www.ams.usda.gov). SFD was measured at the 12th to 13th rib interface perpendicular to the outside surface at a point three-fourths the length of the *longissimus* muscle from its chine bone end. KPH was subjectively estimated as percentage of the carcass weight. In addition, carcass weight (CW) and rib-eye area (REA) were collected as two growth-related phenotypes on these F_2_ animals. CW was determined as the unchilled weight in pounds immediately after harvest before rinsing/washing and chilling. The area of the *longissimus* muscle measured in square inches at the 12th rib interface on the beef forequarter was recorded as REA.

Fatty acid composition in muscle samples was measured according to methodology previously described [17], [19]. In short, approximately 150 mg samples of *longissimus dorsi* muscle tissue were completely saponified with 4.0 ml of 1.18 M KOH in ethanol at 90°C. After about 45 minutes, 2.0 ml of water were added. Cholesterol (CHOL) was extracted with 2.0 ml of hexane, which contained 0.1 mg/ml of stigmasterol as an internal standard for the cholesterol assay. One millilitre of concentrated HCl was added to the original tubes and fatty acids were extracted in 2.0 ml of hexane for fatty acid methyl ester (FAME) preparation using methanolic HCl as a catalyst. The amount of conjugated linoleic acid (CLA) was measured using acid catalysts. FAME data were used to measure the following: saturated fatty acids (SFA)  =  myristic + pentadecanoic + palmitic + heptadecanoic + stearic, monounsaturated fatty acids (MUFA)  =  myristoleic + pentadecenoic + palmitoleic + heptadecenoic + oleic + vaccenic, and polyunsaturated fatty acids (PUFA)  =  linoleic + linolenic. The relative amount of SFA, MUFA and PUFA was defined as SFA  =  (SFA/total fat in 100 g dry meat) ×100%, MUFA  =  (MUFA/total fat in 100 g dry meat) ×100% and PUFA  =  (PUFA/total fat in 100 g dry meat) ×100%, respectively. Three stearoyl-CoA desaturase activities were estimated as R1  =  (14∶1/14∶0) ×100%, R2  =  (16∶1/16∶0) ×100% and R3  =  (18∶1/18∶0) ×100%.

### Gene annotation, mutation discovery and genotyping

A total of 13 genes, including *ABCA1*, *APOA1*, *APOB*, *APOC2*, *APOE*, *LCAT*, *LDLR*, *LIPC*, *LIPG*, *LPL*, *PLTP*, *PON1* and *SCARB1* were selected for the present study. As discussed above, these genes are involved in the RCT pathway. Manual annotation of each gene occurred as follows: First, cDNA sequences of candidate genes were retrieved from the National Center for Biotechnology Information (NCBI) Entrez database. To produce full length cDNA sequences, the retrieved sequences were re-annotated using electronic rapid amplification of cDNA ends (e-RACE) [20]. Next, the full-length cDNA sequence was used to search for genomic DNA contigs against the 7.15X bovine genome sequence database (see the Bovine Genome Resources at NCBI). Primer design was completed using the Primer3 online oligonucleotide design tool [21]. Based on genomic DNA sequences, 37 primer pairs were designed to amplify genetic targets located in 13 genes (Table 1). Approximately 50 ng of genomic DNA from each six Wagyu x Limousin F_1_ bulls was amplified in a final volume of 10 µl that contained 12.5 ng of each primer, 150 µM dNTPs, 1.5 mM MgCl_2_, 50 mM KCl, 20 mM Tris-HCl and 0.25 U of AmpliTaq Gold polymerase (Applied Biosystems, Branchburg, NJ). PCR conditions were as follows: 95°C for 10 minutes, 8 cycles of 94°C for 30 sec, 71°C for 30 sec, and 72°C for 30 sec, followed by 37 cycles of 94°C for 30 sec, 59°C for 30 sec, and 72°C for 30 sec, and completed by an extension step at 72°C for 10 min. PCR amplicons were sequenced on a capillary sequencer by High-Throughput Sequencing Solutions (Seattle, WA). Selected mutations were genotyped in 246 F_2_ animals using the Sequenom iPLEX assay service provided by Genomics Center at University of Minnesota.

**Table 1:** Gene symbols, GenBank references, amplicons and SNPs discovered in the present study.

Symbol	Reference	Amplicon (5′ – 3′)*	SNPs
ABCA1	AAFC03037127	8349 (24) – 8843 (24)	
		7516 (23) – 8091 (23)	
		26751 (23) – 27342 (24)	26841G>T, 27113G>A
	AAFC03121742	35808 (25) – 36322 (23)	
		42352 (24) – 42889 (24)	
		43271 (24) – 43862 (23)	43352C>G, 43466T>C, 43829G>A
		45866 (23) – 46465 (23)	
		72807 (23) – 73325 (23)	73024G>A, 73157C>T
		90523 (23) – 91201 (23)	
		89455 (23) – 90037 (25)	89514T>G
APOA1	AAFC03114751	10221 (23) – 10671 (21)	
		10828 (22) – 11413 (23)	11357G>A
		11797 (24) – 12393 (23)	11919T>G
APOB	AAFC03076821	24087 (24) – 24606 (24)	24295C>T
		38480 (23) – 39240 (23)	38827G>A, 39163G>A
	AAFC03076822	12217 (24) – 12845 (24)	12324T>C
APOC2	AAFC03024850	16125 (21) – 16703 (22)	16569G>A
APOE	AAFC03034452	11376 (24) – 12059 (23)	11400G>A, 11464C>T, 11735G>T
		12364 (23) – 13091 (22)	12439C>T, 12664A>G
		15330 (20) – 16125 (24)	15442C>G, 15532C>T, 15696C>T
LCAT	AAFC03121473	36846 (24) – 37346 (24)	37122G>A
LDLR	AAFC03045894	25 (24) – 621 (23)	
		820 (24) – 1226 (24)	
	AAFC03029857	25894 (22) – 26449 (23)	
LIPC	AAFC03129603	387 (24) – 984 (25)	
		1183 (22) – 1685 (23)	1327C>T, 1499G>A, 1599G>A
LIPG	AAFC03021384	7511 (24) – 8149 (24)	8002G>A
		8189 (23) – 8709 (23)	
LPL	AAFC03023665	34677 (26) – 35241 (25)	
		36200 (24) – 36832 (24)	
PLTP	AAFC03071797	2459 (24) – 2969 (23)	
		13354 (23) – 14092 (23)	13579C>T, 13994G>T
PON1	AAFC03037852	39031 (24) – 39543 (24)	39335G>T
		64143 (23) – 64692 (24)	64207C>T, 64241G>A, 64283A>G
SCARB1	AAFC03038307	17200 (24) – 17666 (23)	17443A>C, 17539C>T
		18769 (22) – 19430 (23)	
	AAFC03119800	6933 (23) – 7429 (21)	

*Number in brackets is the length of forward or reverse primer for the amplicon.

### Data Analysis

The HAPLOVIEW [22] program was utilized to determine degrees of Hardy-Weinberg equilibrium within each marker and linkage disequilibrium between markers within each gene. The association between genotypes and phenotypic traits was evaluated using the GLM (general linear model) procedure of SAS v9.2 (SAS Institute Inc., Gary, NC) based on the following model:
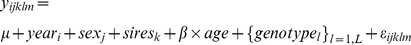
where *y_ijklm_* is phenotypic measurement of a quantitative trait for animal *m*, *year_i_* is the effect of the *i*-th harvest year (*i* = 1,2,3), *sex_j_* is the effect of the *j*-th sex category (*j* = 1,2), *sires_k_* is the effect of the *k*-th sire producing animal *m* (*k* = 1,2,3,4,5,6), *age* is a covariate for age in days of the animal at harvest, and β is the coefficient vector corresponding to the covariate age, *genotype_l_* represents the effects of each genotype at the *l*-th SNP locus, for 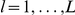, and *ε_ijklm_* is a residual term pertaining to animal *m*. When *L* = 1, the above model reduces to single-marker analysis, and the *P* value is adjusted using Bonferroni correction [23]. Using the same reference population, Jiang and colleagues [24] reported genetic networks associated with 19 complex phenotypes based on a total of 138 genetic polymorphisms derived from 71 known functional genes. After Bonferroni correction, significant (adjusted P<0.05) and suggestive (adjusted P<0.10) genetic markers determined in the present study were merged in the dataset above to identify novel genetic networks involving the RCT pathway. Assignment of quantitative trait modes (QTMs) to each associated marker and linear regression models involving all significant markers for a given phenotype were described previously [23] with minor modifications. Akaike's information criterion (AIC) [25] was used to compare different models each representing a specific genetic network. AIC is a measure of the goodness of fit of an estimated statistical model, panelized by a function of the number of estimated parameters. It is grounded in the concept of entropy, in effect offering a relative measure of the information lost when a given model is used to describe reality and can be said to describe the tradeoff between bias and variance in model construction, or loosely speaking that of accuracy and complexity of the model. Generally, AIC is expressed as:
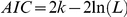
where *k* is the number of parameters, and *L* is the maximized value of the likelihood function for the estimated model. Given a data set, several competing models may be ranked according to their AIC, with the best model having the lowest AIC.

## Results

### SNPs and Haplotypes

As shown in Table 1, the 37 primer pairs used to amplify DNA from the six F1 animals produced a total of 36 single nucleotide polymorphisms (SNPs), including 8 in *ABCA1*, 2 in *APOA1*, 4 in *APOB*, 1 in *APOC2*, 8 in *APOE*, 1 in *LCAT*, 3 in *LIPC*, 1 in *LIPG*, 2 in *PLPT*, 4 in *PON1* and 2 in *SCAB1*, respectively. Nineteen of the 36 SNPs underlined in Table 1 are those selected to form a multiplex SNP set for genotyping by the Sequenom assay design.

**Figure 1:**
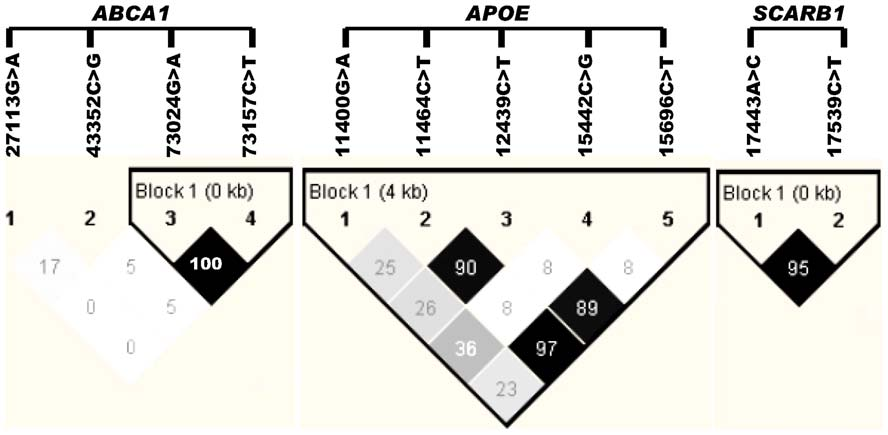
Linkage disequilibrium analysis for markers in the bovine *ABCA1*, *APOE* and *SCARB1* genes. Pairwise linkage disequilibrium relationship for these SNPs are based on r^2^ measurements.

Among 246 animals genotyped, 38 (15.2%) received no calls for *APOA1* - AAFC03114751.1: *c11919T>G*. The *PLTP* - AAFC03071797.1: *g13994G>T* marker was in Hardy-Weinberg disequilibrium (P<0.05). Therefore, these two markers were excluded from further analysis. As a consequence, the HAPLOVIEW analysis was only performed on four markers in *ABCA1*, five SNPs in *APOE* and two markers in *SCARB1* (Figure 1). Strong linkage disequilibrium was detected between 2 of 4 *ABCA1* markers: *73024G>A* and *73157C>T* (*r^2^* = 100%) (Figure 1A), between 3 of 5 *APOE* markers: *11464G>A*, *15442G>C*, *and 15696C>T* (*r^2^* = 89%–97%) (Figure 1B) and between the two *SCARB1* markers, *17443C>A* and *17539:T>C* (*r^2^* = 95%) (Figure 1C). Linkage disequilibrium between the remaining SNPs was low, ranging from 0% to 5% in *ABCA1* and from 8% to 36% in *APOE* gene.

### Single-marker analysis and Bonferroni correction

**Figure 2:**
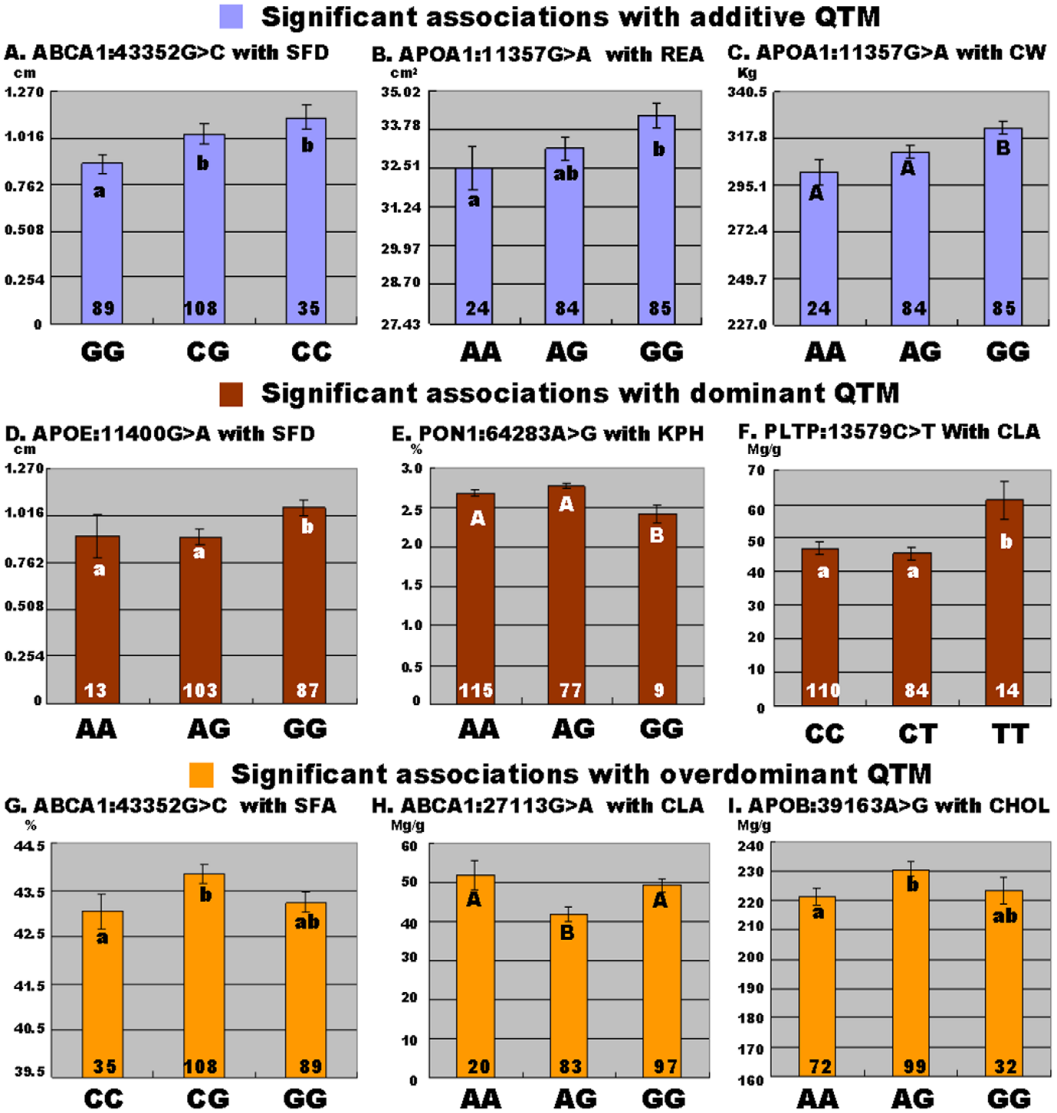
Association of SNP markers with fat decomposition and muscle growth. P values are adjusted by Bonferroni correction. The different capital letters between different genotypes within the same marker means the difference reaches the significance level of adjusted *P*<0.05, while those with difference between genotypes marked by different lowercase letters is suggestive (adjusted *P*<0.10). The same letters between genotypes indicate no suggestive/significant difference (adjusted *P*>0.10). The number within the bars represents the number of animals within each genotype group.

Single-marker analysis revealed seven SNPs significantly associated with nine phenotypes (P<0.05), including *ABCA1* - AAFC03037127.1:*c27113G>A* with CLA and AAFC03121742.1:*g43352C>G* with SFD and SFA, *APOA1* - AAFC03114751.1:*g11357G>A* with CW and REA, *APOB* - AAFC03076821.1:*g39163G>A* with CHOL, *APOE* - AAFC03034452.1:*g11400G>A* with SFD, *PLTP* - AAFC03071797.1:*c13579C>T* with CLA and *PON1* - AAFC03037852.1:*g64283A>G* with KPH, respectively (Figure 2). In other words, two SNPs were found for SFD (Figure 2A and 2D), one for REA (Figure 2B), one for CW (Figure 2C), one for KPH (Figure 2E), two for CLA (Figure 2F and 2H), one for SFA (Figure 2G), and one for CHOL (Figure 2I). After Bonferroni correction, six of these associations remained significant (adjusted *P*<0.05) and three were suggestive (adjusted *P*<0.10).

These nine associations described above could then be classified into three groups, namely, three quantitative trait modes (QTMs): three with additive (Figure 2A–2C), three with dominant (Figure 2D–2F) and three with overdominant effects (Figure 2G–2I). Yet, the QTMs of these markers need to be further confirmed in other populations. When a marker is associated with different phenotypes, it might show the same or different QTMs. For example, *APOA1* - AAFC03114751.1:*g11357G>A* had additive effects on both REA (Figure 2B) and CW (Figure 2C), while *ABCA1* - AAFC03121742.1:*g43352C>G* had an additive effect on SFD (Figure 2A) but an overdominant effect on SFA (Figure 2G). Furthermore, different genetic markers within a gene might contribute to different phenotypes. For example, *ABCA1* - AAFC03037127.1:*c27113G>A* was significantly associated with CLA in an overdominant QTM (Figure 2H), while AAFC03121742.1:*g43352C>G* significantly affected SFD in an additive QTM (Figure 2A) and SFA in an overdominant QTM (Figure 2G).

### Multiple-marker analysis and comparison of models for different genetic networks

**Figure 3:**
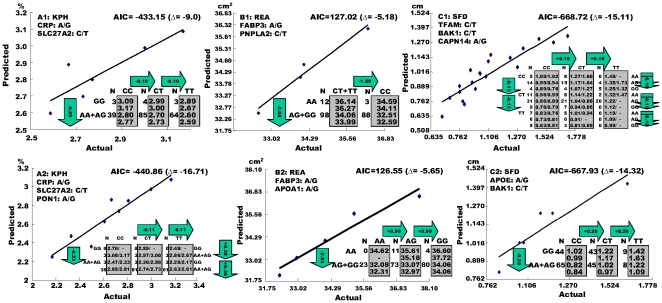
Identification of genetic networks related to RCT pathway via Akaiki Information Criterion based model comparison. A1, B1 and C1 are genetic networks previously reported by Jiang et al. (2009 with permission), while A2, B2 and C2 are newly identified networks in the present study for KPH, REA and SFD, respectively. The x-axis and y-axis represent actual and predicted trait (genotypic) values. The numbers in arrows represent substitution effects of one type of genotypes or allele for another one. In the graph, AIC  =  computed AIC value for a specific model, say A1, and Δ =  the difference of AIC values, say, between model A1 and the base model A0. The AIC values for the three base models, A0, B0 and C0, were −424.15, 132.2, and −653.61, respectively.

The seven markers derived from the RCT pathway were then merged with other markers previously reported by Jiang et al. [24] and combined into a multiple-marker analysis for each trait in attempt to further improve understanding of genetic regulation of fat deposition, fatty acid composition and body growth phenotypes. Several models of genetic networks defined, as shown in Figure 3, and our results suggested that the RCT pathway might be involved in genetic networks for three phenotypes: KPH, REA and SFD. Akaiki Information criterion (AIC) was used to compare different models. The base (null) model (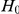) contains the overall mean and systematic effects due to sires, year, sex, and age, but without any SNP/gene effect. In contrast, effects of genes representing possibly different genetic networks are included in alternative, competing models.

For KPH, we previously reported a genetic network involving *CRP* and *SLC27A2* genes, denoted as model A1 (Figure 3). The present study showed that *PON1* gene also contributes to the network for KPH, thus forming a three-gene network, denoted as model A2, for the trait (Figure 3). The AIC value was –424.15 for the base model (A0), and it was −433.15 for model A1 and −440.86 for model A2. The AIC value assigned to each model facilitates ranking of competing models, with the best model having the smallest AIC value. As such, the differences of AIC for model A1 and model A2, as compared with the base model A0, were −9.0 and −16.71, respectively, and the difference of AIC between A1 and model A2 was −7.71. This comparison strongly favors model A2 over model A1, and both models (A1 and A2) over the null model A0.

In our previous study, *FABP3* and *PNPLA2* contributed to formation of a two-gene genetic network for REA, denoted as model B1, with dominant effects for both genes (Figure 3). The present study identified a new genetic network with *FABP3* and *APOA1* (model B2); the former gene with dominant effect while the latter gene with additive effect (Figure 3). AIC-based model selection favored models B1 and B2 almost equally, and both models were favored over the base model. The AIC value was 132.2 for the base model (B0), and it was 127.02 for model B1 and 126.55 for model B2. The difference of AIC between B1 and B0 and between B2 and B0 was −5.18 and −5.65, respectively. Practically, both models B1 and B2 could predict phenotypic performance almost equally well.

The present multiple-marker analysis incorporated markers derived from the RCT pathway and produced a two-gene (*APOE*-*BAK1*) network (model C2) for SFD as an alternative to a three-gene (*TFAM*-*BAK1*-*CAPN1*) network (model C1) identified previously (Figure 3). The new network includes one with dominant effect (*APOE1*) and one with additive effect (*BAK1*), while the all three genes in previously reported network had additive effects. The difference of AIC between model C1 and model C0 and between model C2 and model C0 were −15.11 and −14.32, respectively. Thus, the AIC-based model comparison strongly supported models C1 and C2 over the base model C0, but the difference of AIC between models C1 and C2 were not decisive. Overall, our results showed that the RCT pathway is mainly involved in fat deposition (KPH and SFD) and muscle growth (REA).

## Discussion

Theoretically, lipoprotein pathways contain excellent candidate genes for obesity-related traits because of their direct involvements in transporting cholesterol, triglycerides, and fatty acids to and from peripheral tissues. Other important lipid components are decidedly interconnected with levels of circulating lipoproteins, including saturated fat, which reduces expression of the *LDLR*, leading to increased levels of circulating LDL [26]–[28]. In the present study, we focused on genes involved with RCT pathway and their associations with fat depot storages, fatty acid compositions and overall body growth-related phenotypes using cattle as a model organism. Our results identified 9 significant RCT-pathway associations with CHOL, CLA, CW, KPH, REA, SFA and SFD in a Wagyu x Limousin F_2_ reference population. Obviously, the pathway-based candidate gene approach conducted in this study provides a fast and direct way to determine the genetic variation that underlies complex phenotypes. At the same time, our study also confirmed that genes involved in cholesterol homeostasis are useful targets for investigating obesity in humans [29]–[31].

Up to date, the vast majority of research to understand the genetics of lipoprotein and lipid homeostasis has been directed toward blood lipids. Human ApoE for example, contains two very well known and common SNPs at amino acid positions 112 and 158, which are associated with decreased HDL, increased LDL, and increased plasma cholesterol in circulation [32]–[33]. The direct implications that these *ApoE* mutations have on intramuscular cholesterol are less apparent. Although skeletal muscle cells attain fatty acids via LPL mediated hydrolysis of circulating lipoproteins [34], the lipid compositions of muscle cells and the blood might conceivably be reliant on different mechanisms. This idea is supported by the results of the present study. Of all significant associations determined in the present study, only the *APOB* - AAFC03076821.1:*g39163G>A* was associated with muscle cholesterol levels in an overdominant QTM mode. Furthermore, this marker failed to be incorporated in the genetic network for amount of cholesterol in muscle. This finding is an interesting contrast to the high level of significance found among other lipid traits and might be evidence that intracellular cholesterol is reliant on mechanisms independent of blood pathways.

Noro and Kobayashi [35] hypothesized that the levels of marbling in beef appear to be inversely correlated to HDL levels and directly to LDL. As indicated above, marbling reflects the dispersion of fat within the muscle, which is subjectively measured as intramuscular fat stored in the *longissimus* muscle. Unfortunately, our present study could not provide any evidence to support the hypothesis: none of the sequence variations in genes associated with the RCT pathway was associated with variation in marbling. In contrast, *APOA1* - AAFC03114751.1:*g11357G>A* impacted REA, which is a measurement of the size of the *longissimus* muscle. This means that *APOA1* gene might be involved in regulation of muscle growth. In particular, both *APOA1* and *FABP3* genes combine to affect REA. Just recently, Teltathum and Mekchay [36] reported that APOA1 and FABP3 are two of five proteins that are expressed in chicken muscle in an age dependent fashion. The authors observed that the expression levels of APOA1 and FABP3 proteins were negatively correlated with chicken aging (p<0.05). This indicates that the genetic network established in the present study for REA makes sense, because both *APOA1* and *FABP3* genes are involved in muscle development and growth.

In summary, our present study revealed that sequence variations of genes in the RCT pathway are associated with KPH, REA and SFD. Both KPH and SFD are phenotypes related to fat deposition, while REA is connected to muscle growth. From the livestock production point of view, the SNPs evaluated in the present study are strong candidates to join existing panels for marker-assisted selection of meat quality phenotypes in beef cattle. The markers identified in the present study might also have implications beyond the field of animal breeding and improvements and be directive in RCT pathway-related disease research.
